# The genetic mechanism of selfishness and altruism in parent-offspring coadaptation

**DOI:** 10.1126/sciadv.aaw0070

**Published:** 2020-01-03

**Authors:** Min Wu, Jean-Claude Walser, Lei Sun, Mathias Kölliker

**Affiliations:** 1 Department of Environmental Sciences, Zoology and Evolution, University of Basel, Basel, Switzerland.; 2 Department of Environmental Systems Science, Genetic Diversity Centre (GDC), ETH Zürich, Zürich, Switzerland.; 3 Institute of Integrative Biology, ETH Zürich, Zürich, Switzerland.

## Abstract

The social bond between parents and offspring is characterized by coadaptation and balance between altruistic and selfish tendencies. However, its underlying genetic mechanism remains poorly understood. Using transcriptomic screens in the subsocial European earwig, *Forficula auricularia*, we found the expression of more than 1600 genes associated with experimentally manipulated parenting. We identified two genes, *Th* and *PebIII*, each showing evidence of differential coexpression between treatments in mothers and their offspring. In vivo RNAi experiments confirmed direct and indirect genetic effects of *Th* and *PebIII* on behavior and fitness, including maternal food provisioning and reproduction, and offspring development and survival. The direction of the effects consistently indicated a reciprocally altruistic function for *Th* and a reciprocally selfish function for *PebIII*. Further metabolic pathway analyses suggested roles for *Th-*restricted endogenous dopaminergic reward, *PebIII-*mediated chemical communication and a link to insulin signaling, juvenile hormone, and vitellogenin in parent-offspring coadaptation and social evolution.

## Teaser

Two genes coordinate selfishness and altruisim, and more than 1600 genes are associated with parenting in mothers or offspring.

## INTRODUCTION

Parents and offspring influence each other’s behavior and evolutionary fitness through reciprocal interactions (*1*). As an altruistic trait, parental care is beneficial to the survival and development of offspring but is costly for the parents, while selfish parents favor their lifetime fecundity at the expense of their offspring’s fitness (*1*). Offspring are often tacitly regarded as passive recipients of parental care, but in reality, they actively demand care and influence their parents’ behavior and reproduction (*1*). Evolutionary theory predicts a tension between selfishness and altruism and genetic conflict between parents and offspring over parental investment due to their incomplete relatedness (*1*, *2*). Theoretical models predicted that natural selection should favor resolved conflict and coadapted parent and offspring (*3*), a state characterized by (i) a compromise between the evolutionary interests of parent and offspring, (ii) balanced altruistic and selfish genetic tendencies, and (iii) well-coordinated behavioral interactions between them (*1*, *2*). To date, studies on conflict resolution and coadaptation have focused on phenotypes rather than genes (*3*), and studies on the molecular basis of parenting focused on genes expressed merely in parents and lacked causal evidence (*4*–*7*).

To identify genes underlying parent-offspring coadaptation and to study their altruistic and selfish function during social interaction, a combination of these former approaches is required. In addition, offspring should be regarded as active players. Genes expressed in offspring and affecting parental care, for example, through effects on solicitation behaviors, should be studied.

In this study, we used the European earwig, *Forficula auricularia*, as an experimental system. *F. auricularia* is a nonmodel insect species with facultative posthatching maternal care, enabling behavioral manipulations with and without mother-offspring contact, without detrimental effects on offspring. Females produce one or two clutches over their lifetime and provide food (see movie S1) and protection to their young nymphs (*8*, *9*). Mothers influence the behavior, development, and survival of their nymphs, and the nymphs, in turn, influence the behavior and future reproduction of their mothers, for instance, by chemical communication (*2*, *10*, *11*).

**Fig. 1:**
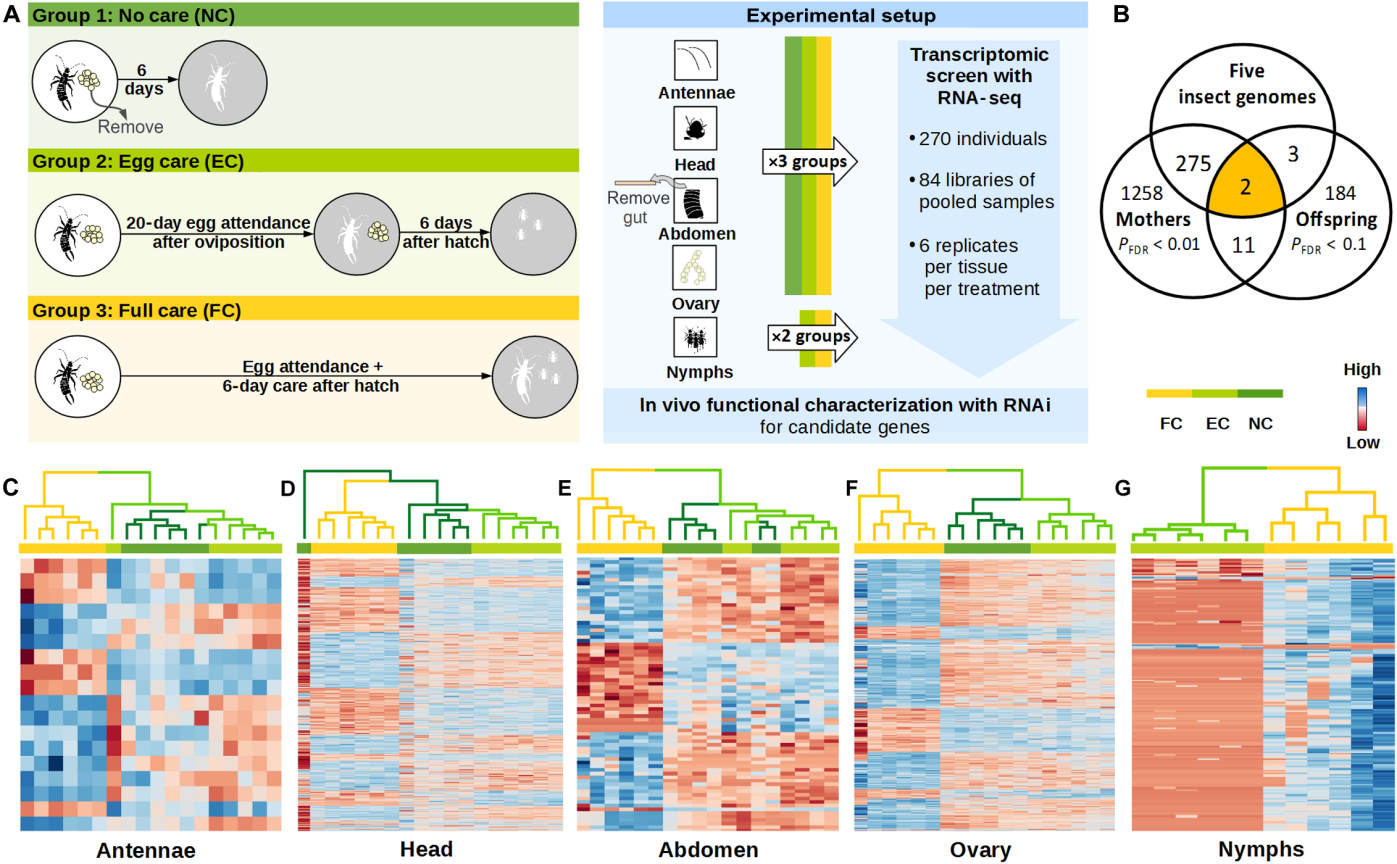
Experimental design and expression heat map. (**A**) The transcriptomic screen for candidate genes underlying parent-offspring coadaptation was based on experimental manipulation of the presence and absence of egg attendance and posthatching parental care: No care (NC), eggs were removed upon the completion of oviposition. No nymph sample was used because of insufficient hatching success of untended eggs (fig. S1 and table S1). Egg care (EC), mothers tended their eggs for 20 days and were then sampled shortly before eggs hatched (*2*). Nymphs were kept for 6 days without tending females before they were sampled. Full care (FC), mothers tended their eggs until hatching and cared for their nymphs until 6 days after hatching when both were sampled. Antennae, head, abdomen, and ovaries from mothers and the whole body of nymphs were sampled in each treatment. On the basis of this screen, the expression of candidate genes was manipulated using in vivo RNAi to assess their causal effects on behavior and components of evolutionary fitness. (**B**) Venn diagram illustrating that only two genes with confirmed insect origin were differentially coexpressed in mothers and offspring when they behaviorally interacted according to our selection criterion (see main text). (**C** to **G**) Heat maps of differentially expressed genes in different tissues from mothers and the nymphs (*P*_FDR_ < 0.01). Rows are genes, columns are samples. Samples were clustered according to expression patterns. We color-coded each sample in the horizontal bar above each panel and the clustering trees according to its treatment. FC samples are in yellow; EC samples are in light green, and NC samples are in dark green.

Our experimental approach was to first broadly quantify differential gene expression between manipulated treatments with or without parental care in both mother and offspring in an RNA sequencing (RNA-seq) experiment (Fig. 1A). We then identified candidate genes on the basis of these data and characterized their social function in an RNA interference (RNAi) knockdown experiment. As a criterion for selecting candidate genes, we used the differential coexpression of the same gene between experimental treatments in earwig mothers and nymphs in the RNA-seq data. Although coadaptation may occur between different sets of genes expressed in parents or offspring, it is of limited scope in the absence of physical linkage (*3*). The same genes affecting different traits in parents and offspring, a form of pleiotropy, provide scope for much tighter coadaptation (*3*). Mechanistically, this differential coexpression could be due to specific alleles in the cis-regulatory region of the pleiotropic gene reflecting a state of coadaptation.

We hypothesized that candidate genes underlying coadaptation should have the following signatures: (i) Gene expression changes in the parent or offspring when they behaviorally interact, with the strongest candidates showing differential coexpression of the same gene, irrespective of the direction of expression difference being parallel or antagonistic. (ii) For these candidate genes to be biologically relevant, an experimentally manipulated change in the expression level in the parent or offspring should influence behavior or fitness of self and the other via direct and indirect genetic effects (DGE and IGE), respectively (*12*, *13*). (iii) When expressed, a gene with a selfish function should be beneficial to self and potentially harmful to the other, and a gene with an altruistic function, when expressed, should be beneficial to the other and costly to self. Here, we found two genes fulfilling these criteria in *F. auricularia*, and we discuss their potential roles in the evolution of parent-offspring interaction and social evolution.

## RESULTS

### Transcriptomic screen for candidate genes underlying coadaptation via RNA-seq

To screen for candidate genes, we experimentally manipulated whether mothers attended their eggs and whether they cared for and socially interacted with their hatched nymphs. To this end, we established three treatments, no care (NC), egg care (EC), and full care (FC) (Fig. 1A). The FC treatment differed from the EC treatment in the occurrence of social interaction between mothers and nymphs, and the EC and NC treatments differed in egg attendance. Ninety females with their clutches were randomly assigned to the three treatments, with 30 per treatment. To detect tissue-specific expression, we separately sequenced the transcriptome of four maternal tissues including antennae, head, abdomen, and ovaries and the whole body of nymphs with RNA-seq. All nymphs were sampled on day 6 after hatching to avoid confounding variation caused by age or developmental stage.

A total of 138 gigabytes of Illumina HiSeq data were generated from 84 libraries and mapped to a previously published earwig transcriptome (*14*). Each maternal library was a pool of the same tissue from five individuals. Each nymph library was a pool of five clutches with three individuals each. NC nymphs were not used because of low hatching success (fig. S1 and table S1).

A total of 1547 genes in at least one maternal tissue and 114 genes in nymphs were differentially expressed between the FC and EC treatments (*P*_FDR_ < 0.01) (data files S1 to S5). All of the FC samples were well clustered on the basis of the expression of these genes. Samples from NC and EC treatments were more similar to each other (Fig. 1, C to G). This pattern was true for all maternal tissues and nymphs, indicating consistently and broadly altered gene expression when mothers and their offspring behaviorally interacted.

#### Identification of candidate genes

As pointed out above, the most stringent condition for candidate genes underlying coadaptation in our transcriptomic data is the differential coexpression between FC and EC in mothers and nymphs during posthatching maternal care. The NC treatment was not used for candidate gene selection because its difference to the FC treatment could result from both posthatching maternal care and egg attendance, and no offspring data were available. Coadaptation theory predicts stronger coadaptation when the same genes alter their expression in parent and offspring during their social interaction, which may facilitate well-coordinated parenting by enhancing the phenotypic match between parent and offspring (*3*).

At the significance level corrected for false discovery rate (FDR) < 0.01, we found no insect gene with differential coexpression between the FC and EC treatment in mothers and offspring. This was partly due to the fact that the transcriptome of nymphs was based on whole bodies, obscuring any potential tissue-specific expression. Thus, our measure of differential expression in nymphs was less precise, and our statistical power for their detection was lower. To expand the number of detected genes, we relaxed the significance threshold for nymphs to be less stringent with *P*_FDR_ < 0.1. We found 13 genes differentially expressed between FC and EC treatments in mothers with *P*_FDR_ < 0.01 and in offspring with *P*_FDR_ < 0.1. We then used five insect genomes to filter out genes with insect origin. These genomes were previously used to annotate a published earwig transcriptome (*14*). They comprise two eusocial species, the honey bee (*Apis mellifera*) and the leaf-cutting ants (*Acromyrmex echinatior*), and three solitary species, the fruit fly (*Drosophila melanogaster*), the red flour beetle (*Tribolium castaneum*), and the jewel wasp (*Nasonia vitripennis*). Only two of the differentially coexpressed genes were present in at least one of the five insect genomes. We further checked the 11 remaining genes manually. Two had unknown origin and function, and nine were not from insects. Thus, the two genes, with confirmed insect origin and evidence of differential expression between the FC and EC treatment in both mothers and offspring during posthatching parental care, were selected as our candidates (Fig. 1B). One of them, Contig4258 in the published earwig transcriptome (*14*), is homologous to the *D. melanogaster Th* gene. The other, Contig29301, is homologous to the *PebIII* gene. *Th* encodes tyrosine hydroxylase, the first and rate-limiting enzyme in the dopamine synthesis pathway (*15*). *PebIII* encodes ejaculatory bulb protein III, an odorant-binding protein (OBP) (*16*).

#### Th and other genes related to dopamine

**Fig. 2:**
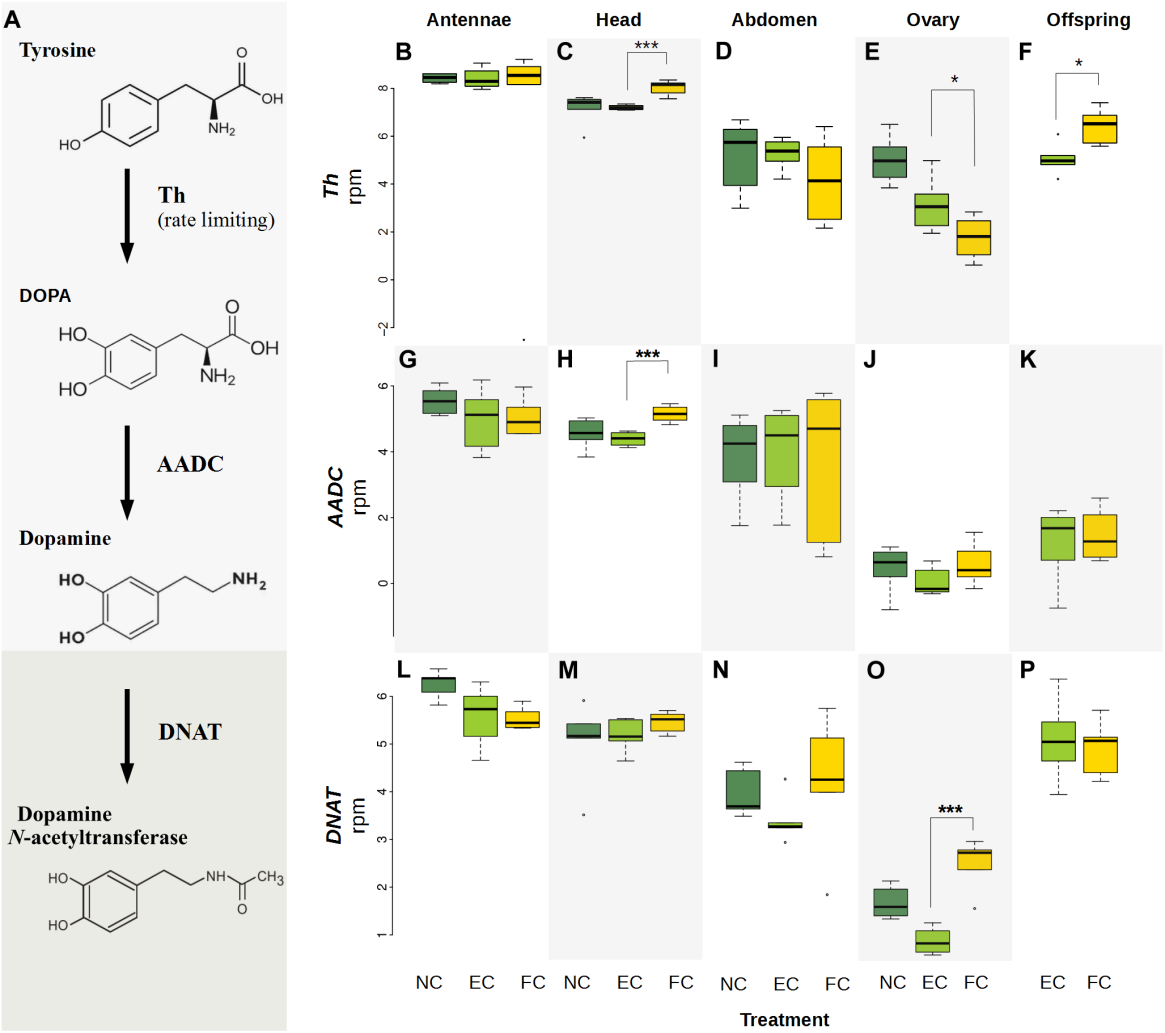
Differential expression of *Th* and other dopamine-related genes in maternal tissues and in offspring. (**A**) Illustration of a known dopamine synthesis and degradation pathway catalyzed by tyrosine hydroxylase (Th) as the rate-limiting enzyme as well as DOPA decarboxylase (AADC) and DNAT (*15*, *17*). (**B** to **F**) Differential expression of *Th* across treatments. (**G** to **K**) Differential expression of *AADC*. (**L** to **P**) Differential expression of *DNAT*. The *y* axes are normalized read counts. Treatments are color-coded, with NC in dark green, EC in light green, and FC in yellow. The box plots are shown with medians, interquartile range (box), and 1.5× interquartile range (whiskers). *P*_FDR_ = 0.053 for *Th* differential expression between FC and EC in ovaries, *P_FDR_ = 0.056 in offspring and ***P_FDR_ < 0.001.

Compared with the EC treatment, *Th* expression was higher in the FC mother’s head (*P*_FDR_ < 0.0001; Fig. 2C) and in her nymphs (*P*_FDR_ = 0.056; Fig. 2F). In addition, we found higher expression of 3,4-dihydroxyphenylalanine (DOPA) decarboxylase [AADC (aromatic l-amino acid decarboxylase); Contig5494; *P*_FDR_ = 0.00018; Fig. 2H], the downstream enzyme of tyrosine hydroxylase that catalyzes the last step of dopamine synthesis (*15*), in the FC mothers’ head. Together, these results suggested enhanced dopamine activity in the head, where the central nervous system is located. Furthermore, *Th* showed a trend of reduced expression in the ovaries of FC mothers compared to EC mothers (*P*_FDR_ = 0.053; Fig. 2E). This was in agreement with an elevated expression of dopamine *N*-acetyltransferase (DNAT; Contig14038; *P*_FDR_ < 0.0001; Fig. 2M), an enzyme for dopamine degradation (*17*), in the ovaries of FC mothers compared to EC mothers. These results suggested decreased activity of dopamine in the ovaries during posthatching care. Dopamine is a well-studied neurohormone with a conserved function as neurotransmitter in the reward system and for associative learning from insects to mammals (*18*, *19*). Dopamine also functions as gonadotropin to stimulate reproduction in various insect species including fruit flies, bees, and ants (*20*–*22*). Thus, the fact that earwig mothers suppress their reproduction during parenting (*2*) may be partly regulated by the antagonistic expression of *Th* and dopamine in the head and ovaries. Conversely, the enhanced expression of *Th* in the mother’s heads and in her nymphs during posthatching care suggested a role of a mutual dopaminergic reward in maintaining the social bond between them.

#### PebIII and other genes related to chemical communication

**Fig. 3:**
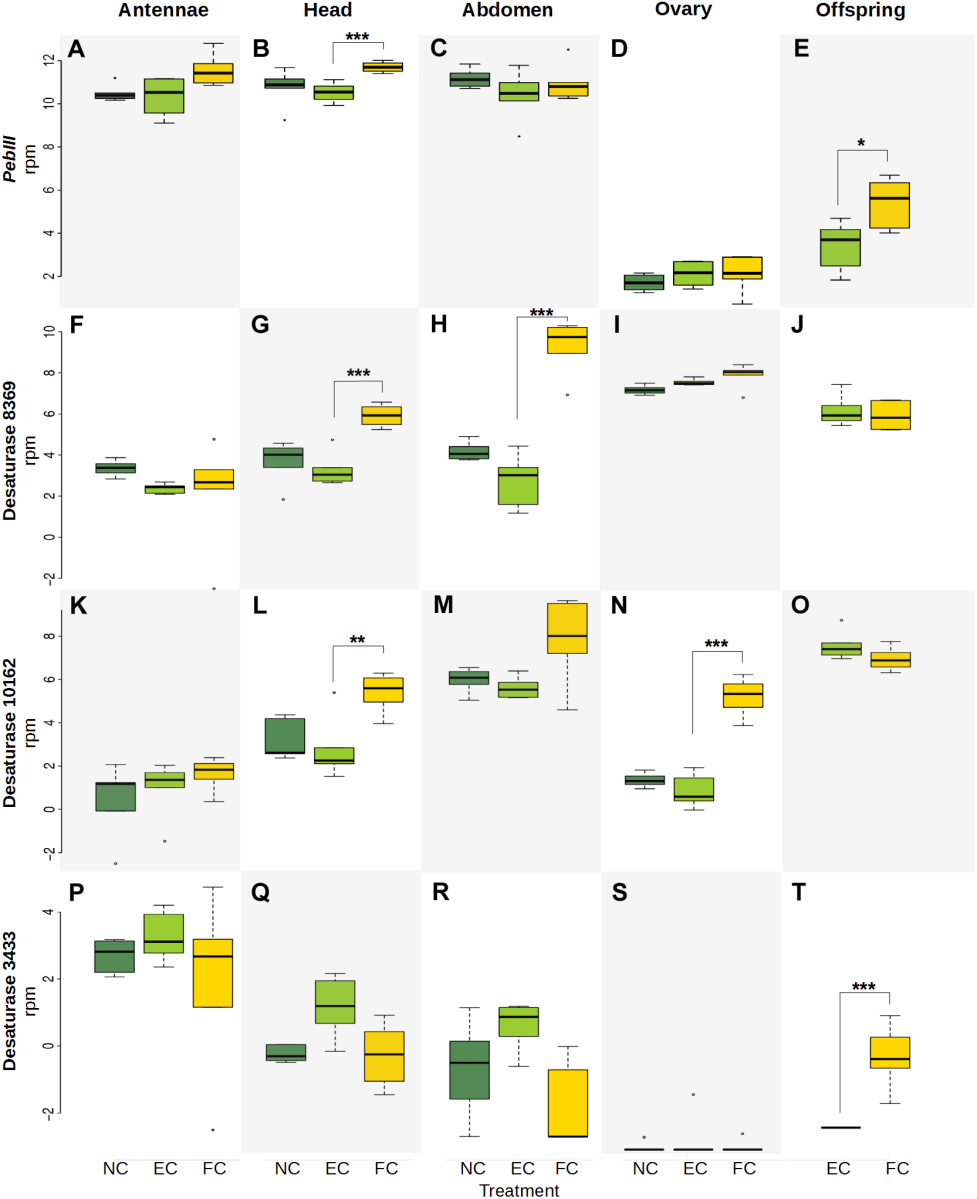
Differential expression of *PebIII* and acyl-CoA desaturase genes in maternal tissues and in offspring. (**A** to **E**) Differential expression of *PebIII*. (**F** to **T**) Differential expression of acyl-CoA desaturase genes potentially for CHC synthesis and chemical communication. To distinguish multiple transcripts of desaturase genes, the corresponding contig ID is labeled next to each gene name. *y* axes are normalized read counts. Treatments are color-coded with NC in dark green, EC in light green, and FC in yellow. The box plots are shown with medians, interquartile range (box), and 1.5× interquartile range (whiskers). *P_FDR_ = 0.034, **P_FDR_ < 0.01, and ***P_FDR_ < 0.001.

Compared with the EC treatment, *PebIII* expression in mothers was enhanced in the FC mother’s head (*P*_FDR_ < 0.0001; Fig. 3B) and in her nymphs (*P*_FDR_ = 0.034; Fig. 3E). Given the putative function of *PebIII* as OBP, its involvement in parent-offspring communication and the perception of chemical cues such as cuticular hydrocarbons (CHC) (*11*) is conceivable. The increased expression of *PebIII* in the FC treatment may refer to enhanced olfactory sensitivities when mothers and nymphs interact. A link to chemical communication was further supported by the higher expression of a few genes homologous to acyl–coenzyme A (CoA) desaturase previously shown to be involved in CHC synthesis (*23*) in FC mother’s head, abdomen, and ovaries (Contig8369 and Contig10162, *P*_FDR_ < 0.01 for all; Fig. 3, G, H, L, and N) and in FC nymphs than EC treatment (Contig3433, *P*_FDR_ < 0.0001; Fig. 3T).

**Fig. 4:**
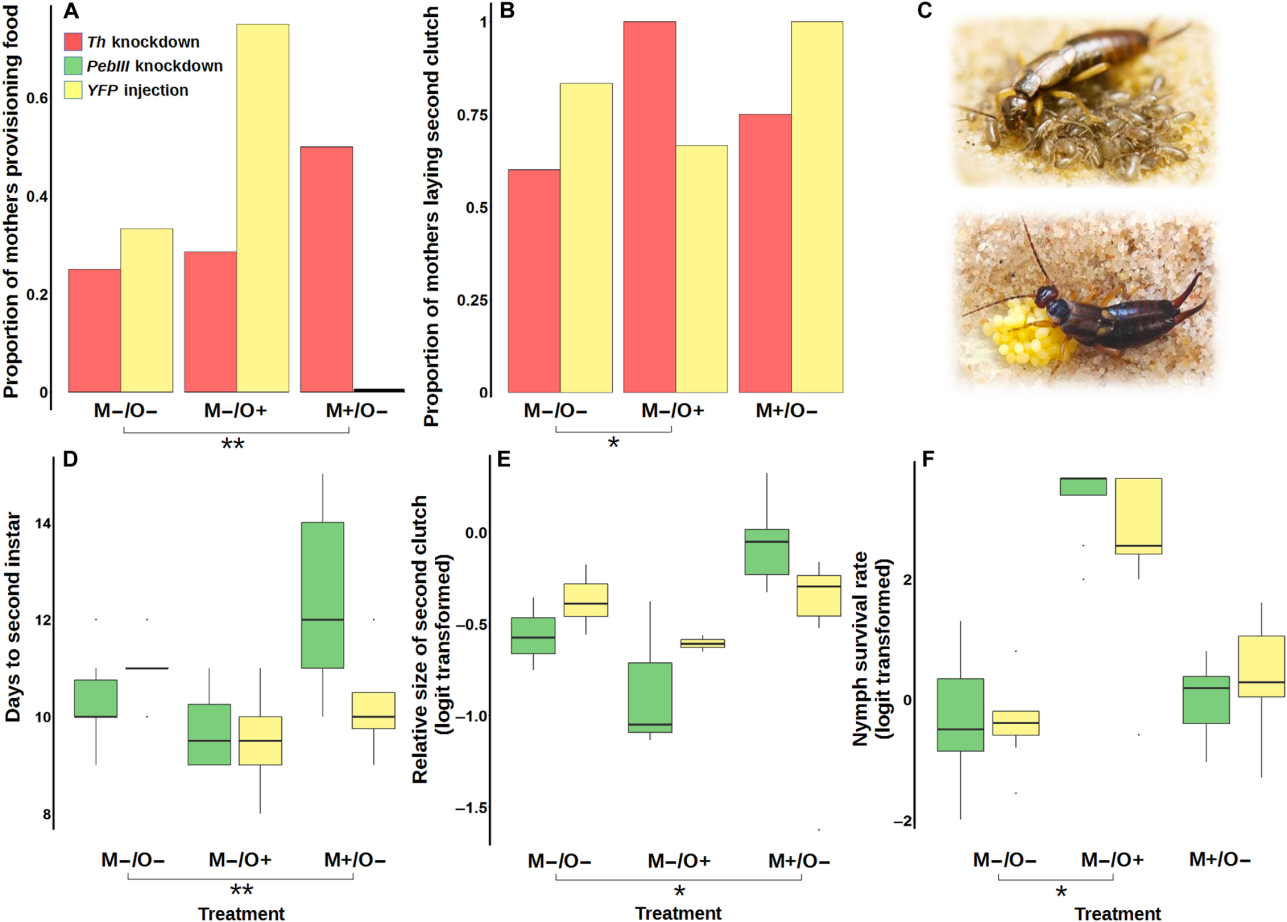
Effects of *Th* and *PebIII* knockdown on behavior and measures of fitness in earwig mothers and nymphs. (**A** and **B**) Results for *Th* knockdown. (**C**) Pictures of an earwig mother tending hatched nymphs (top) (photo credit to J. Meunier) and tending her eggs (bottom). (**D** to **F**) Results for *PebIII* knockdown. In (E), the relative size of the second clutch was calculated on the basis of the first and second clutch egg numbers. In (A) and (B), frequencies are shown, Note that zero observed provisioning in the M+/O− *YFP* treatment means that no instance of provisioning was observed during the observation sessions (see Materials and Methods). (D to F) Box plots with median, interquartile range (box), and 1.5× interquartile range (whiskers). Target genes were knocked down in three treatments: only in mothers (M−/O+), only in offspring (M+/O−), and in both (M−/O−). Three corresponding *YFP* treatments were used to control for the injection of exogenous double-stranded RNA. To test the specific effects of target gene knockdown over and above effects of injection of double-stranded RNA, we used a GLM for statistical analysis with the main effects gene, maternal treatment, and offspring treatment (table S2). Significant gene–by–maternal treatment interaction or gene–by–offspring treatment interactions indicate a social function of a target gene, which are labeled with asterisks; *P < 0.05 and **P < 0.01.

### Social function of *Th* and *PebIII* characterized by in vivo RNAi

To characterize the social function of the two candidate genes in vivo, we knocked down the expression of *Th* or *PebIII* in mothers (M−/O+), in the offspring (M+/O−), or in both (M−/O−) using RNAi. Note that the “−” refers to the injection of double-stranded RNA. To control for confounding side effects of exogenous double-stranded RNA injection, we also established three corresponding sham treatments of *YFP* (yellow fluorescent protein gene) with injection in mothers (M−/O+), in the offspring (M+/O−), or in both (M−/O−). Seventy earwig mothers with their overall 1363 nymphs were assigned randomly to these treatments. The specificity of knockdown was validated by reverse transcription quantitative polymerase chain reaction (RT-qPCR) in the mothers’ head and the whole body of nymphs (Wilcoxon test, *P* < 0.05; fig. S2).

Sham treatments injecting double-stranded RNA of an exogenous gene such as *YFP* were a necessary control because we expected that the injection of RNA per se would influence survival, behavior, development, or reproduction. To statistically control for these confounding effects and demonstrate the specific effects of knockdown of the target gene, we compared target gene injection treatments with the corresponding *YFP* sham treatments using generalized linear models (GLMs). In the GLMs, we defined the fixed main effects “gene” for the overall difference between target gene and *YFP* injection, “maternal treatment” for the overall difference between injected and untreated females, and “offspring-treatment” for the overall difference between injected and untreated nymphs on the basis of our experimental design. To determine the function of a target gene, the effects of maternal or offspring injection should depend on whether it was due to the injection of double-stranded RNA of target gene or *YFP*. In other words, only the interaction between gene and maternal treatment or the interaction between gene and offspring treatment resulted from the GLM analysis tested the specific function of a target gene on a dependent trait. Whether the gene operated via a DGE or an IGE depended on whether the effect was in the focal individual or in its social partner.

#### Th: A gene reciprocally promoting altruism

*Th* knockdown in mothers affected food provisioning (Fig. 4A and table S2, gene–by–maternal treatment interaction, *P* = 0.0043, GLM). This effect arose from less observed food provisioning in *Th*-knockdown mothers than in *YFP*-injected mothers in the M−/O− treatment and more observed food provisioning in *Th*-untreated mothers than *YFP*-untreated mothers in the M+/O− treatment. M−/O− and M+/O− differ in maternal injections, but the offspring were injected in both treatments. Thus, comparing M−/O− to M+/O−, *Th* knockdown in mothers resulted in a relative reduction of food provisioning. Put differently, *Th* expression in mothers enhanced food provisioning via a DGE, over and above any effect due to the injection of exogenous double-stranded RNA.

Moreover, *Th* knockdown in nymphs affected their mothers’ likelihood to produce a second clutch (Fig. 4B and table S2, gene–by–offspring treatment interaction, *P* = 0.037, GLM). The mothers of *Th-*knockdown offspring tended to be less likely to produce a second clutch than the mothers of *YFP*-injected offspring in the M−/O− treatment, and the mothers of *Th-*untreated offspring tended to be more likely to lay a second clutch than the mothers of *YFP*-untreated offspring in the M−/O+ treatment. Thus, comparing M−/O− to M−/O+, *Th* knockdown in offspring reduced the likelihood of maternal future reproduction, or in other words, *Th* expression in offspring enhanced the likelihood of maternal future reproduction through an IGE.

Enhanced food provisioning is a typical parental behavior that elevates the fitness of offspring at a cost to mothers (*1*, *8*). Thus, elevated expression of *Th* in mothers induced a maternal behavior that is beneficial to offspring and costly for themselves. Increased maternal future reproduction enhances maternal lifetime fecundity, but from the perspective of the current nymphs inducing this effect, it is at the cost of reduced received care, as formerly demonstrated in experimental evolution experiments in this species (*2*). Hence, in both mothers and nymphs, higher expression of *Th* enhanced the fitness of the other at a potential expense of self, which is consistent with our prediction of an altruistic gene.

#### PebIII: A gene reciprocally promoting selfishness

*PebIII* knockdown affected offspring developmental rate (Fig. 4D and table S2, gene–by–maternal treatment effect, *P* = 0.0013, GLM). The offspring of *PebIII-*knockdown mothers reached the second instar in fewer days than the offspring of *YFP-*injected mothers in the M−/O− treatment, while the offspring of *PebIII-*untreated mothers took more days to reach the second instar than the offspring of *YFP*-untreated mothers in the M+/O− treatment. Therefore, comparing M−/O− to M+/O−, *PebIII* knockdown in mothers caused faster offspring development, or in other words, *PebIII* expression in mothers delayed offspring development through an IGE.

Furthermore, *PebIII* knockdown in mothers affected the size of their second clutch (Fig. 4E and table S2, gene–by–maternal treatment interaction, *P* = 0.017, GLM). The *PebIII* knockdown mothers tended to have smaller relative size of their second clutch than the *YFP*-injected mothers in the M−/O− treatment, but the *PebIII*-untreated mothers had larger relative size of second clutch than the *YFP*-untreated mothers in the M+/O− treatment. Thus, comparing M−/O− to M+/O−, *PebIII* knockdown in mothers had the effect to reduce the size of the second clutch. In other words, *PebIII* expression in mothers increased their own investment in future reproduction through a DGE.

Last, *PebIII* knockdown in nymphs influenced their own survival (Fig. 4F and table S2, gene–by–offspring treatment, *P* = 0.037, GLM). The *PebIII* knockdown offspring showed no difference of survival compared to the *YFP*-injected offspring in the M−/O− treatment, but the *PebIII-*untreated offspring survived better than the *YFP*-untreated offspring in M−/O+ treatment. Hence, comparing M−/O− to M−/O+, it was *PebIII* knockdown in offspring that led to relatively stronger reduction of survival than YFP injection. In other words, *PebIII* expression in offspring enhanced their own survival through a DGE.

In all three cases, individuals that expressed *PebIII* gained benefits for themselves but partially harmed the fitness of the other. For instance, the enhanced future reproduction of mothers and the better survival of offspring were both influenced by their own expression of this gene, but the delayed offspring development, potentially having negative consequences, was influenced by maternal expression through an IGE. These results are consistent with our prediction for a selfish gene.

### Differentially expressed genes for insulin signaling, juvenile hormone, and vitellogenin regulation

That *Th* influenced the mothers’ likelihood of producing a second clutch might be partially explained by the *Th*-restricted dopamine because dopamine functions not only as a neurotransmitter but also as gonadotrophin in insects (*18*–*22*), but how *PebIII* influenced reproduction in mothers through a DGE is less straightforward. *PebIII* is indirectly linked to the yolk protein vitellogenin (Vg) and juvenile hormone (JH) via apolipophorin and lipophorin (*24*, *25*). Vg and JH are well known to regulate female reproduction and interact with insulin signaling in many insects (*26*–*30*). We thus further explored the expression of genes related to this pathway in earwig mothers using our RNA-seq data.

**Fig. 5:**
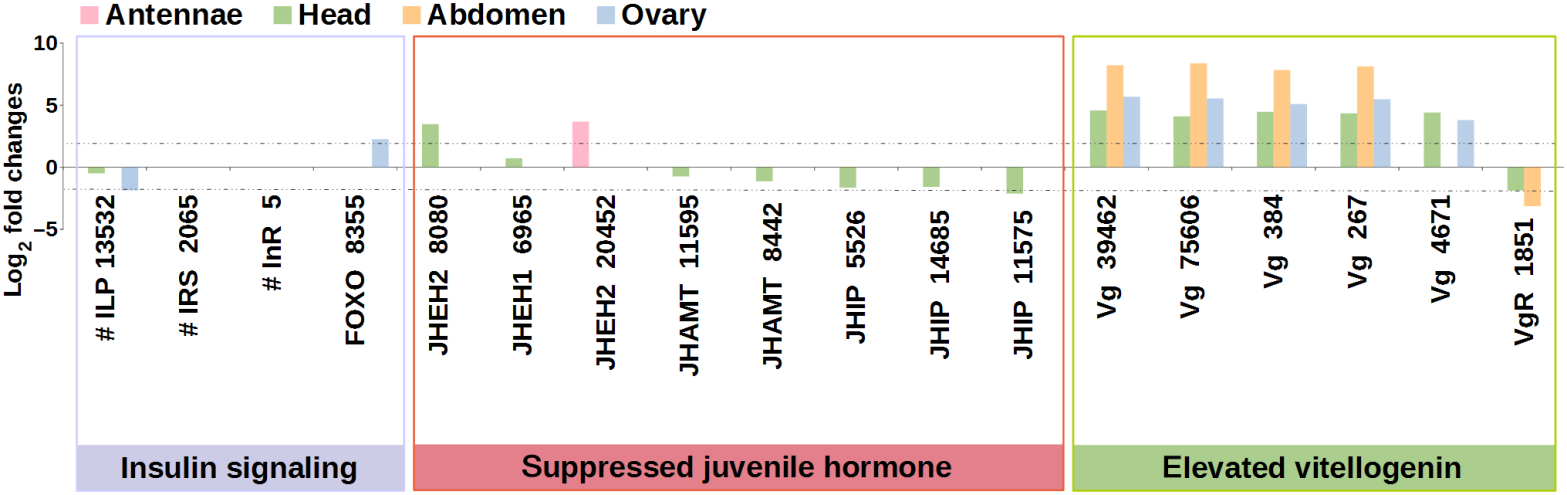
Differential expression of genes related to insulin signaling, JH, and Vg. Bars above zero indicate enhanced expression in FC mothers compared to EC mothers in the RNA-seq experiment, while bars below zero indicate reduced expression. Dotted lines indicate thresholds for 2 and −2 log_2_ fold changes. ILP, insulin-like peptide; IRS, insulin receptor substrate; InR, insulin receptor; FOXO, forkhead domain transcription factor; JHIP, JH ineducable protein; VgR, Vg receptor. To distinguish multiple transcripts of the same gene, the contig ID is labeled next to the corresponding gene name. # ILP showed a trend of differential expression with *P*_FDR_ = 0.065 in the mothers’ head and *P*_FDR_ = 0.058 in the ovaries, but there was no expression difference found for IRS or InR. *P*_FDR_ < 0.01 for all the other genes.

Compared to EC mothers, we found evidence for reduced insulin signaling and JH but elevated Vg in FC mothers providing posthatching care. With regard to JH, the expression of JH epoxide hydrolase (JHEH), an enzyme degrading JH (*31*), was enhanced in their head (Contig8080, *P*_FDR_ < 0.0001; Contig6965, *P*_FDR_ = 0.00019; Fig. 5) and antennae (Contig20452, *P*_FDR_ = 0.00041; Fig. 5). Conversely, the expression of JH acid *O*-methyltransferase (JHAMT), a key enzyme converting inactive precursors of JH to active JH in insects (*32*), was reduced in their head (Contig11595, *P*_FDR_ = 0.00064; Contig8442, *P*_FDR_ < 0.0001; Fig. 5). Last, JH-inducible proteins were down-regulated in their head (Contig5526, Contig14685, and Contig11575, *P*_FDR_ < 0.0001 for all; Fig. 5). These results are consistent with a previous earwig study showing that a low JH titer was associated with maternal care for nymphs in mothers (*33*). With regard to Vg, five contigs of Vg genes were up-regulated in the head, abdomen, and ovaries of FC mothers (Contig39462, Contig75606, Contig384, Contig267, and Contig4671, *P*_FDR_ < 0.0001 for all; Fig. 5), but the Vg receptor was down-regulated (Contig1851, *P*_FDR_ < 0.0001 in the head and *P*_FDR_ = 0.0012 in the abdomen; Fig. 5). Gonadotropic functions of JH and Vg are well known in insects (*26*, *27*, *29*, *30*) and are related to caste determination and division of labor in eusocial species such as the honey bee (*A. mellifera*) (*27*). Vg is also related to brood care in the burying beetle (*Nicrophorus vespilloides*), honey bee, and the ant (*Temnothorax longispinosus*) (*26*, *34*–*36*). The antagonistic regulation between JH and Vg previously thought to be unique in honey bee (*27*) is comparable to our findings in the subsocial European earwigs and the burying beetles (*26*), suggesting the role of antagonistic JH-Vg regulation in posthatching parental care and social evolution.

With regard to insulin signaling, an association with reproductive asymmetries between castes was described in eusocial ants (*28*). In the present study, we found a trend of decreased expression of the insulin-like peptide gene *ILP* in FC compared to EC mothers (Contig13532, *P*_FDR_ = 0.065 in the head and *P*_FDR_ = 0.058 in the ovaries; Fig. 5). In addition, the *FOXO* transcription factor, which is known to suppress insulin signaling (*37*), was significantly higher in FC than EC mothers’ ovaries (Contig8355, *P*_FDR_ = 0.00091; Fig. 5). These results suggested a role for insulin signaling in mediating posthatching care in the subsocial earwigs.

### Vasopressin/oxytocin-like peptide

Last, oxytocin and vasopressin are neuropeptides that were previously shown to be associated with parental care in mammals (*7*, *38*). We found an earwig homolog (Contig9205) of the *Tribolium* gene, according to their translated protein sequence comparison, for the receptor for the insect vasopressin/oxytocin-like peptide (*39*). However, in our experiment, no differential expression was found between FC and EC mothers (*P*_FDR_ = 0.50), confirming previous findings that vasopressin/oxytocin-like peptide may function differently in insects and mammals (*39*).

## DISCUSSION

In this study, we found more than 1600 genes in earwig mothers and offspring that were differentially expressed in association with posthatching maternal care. Among these, *Th* and *PebIII* showed the hypothesized signatures of coadaptation, with differential coexpression between treatments in both mothers and offspring during posthatching care when they socially interact, and causal effects on behavior and measured fitness that indicated altruistic and selfish tendencies, respectively. Our results demonstrate that genes expressed in offspring are active players shaping maternal behavior and reproduction. Whether other differentially expressed genes are related to mother and nymph behavior and whether they may be shaped by coadaptation require further study.

Our findings are also of direct interest to better understand the molecular link between maternal care and worker care in insects and, hence, the evolutionary transition from parenting to eusociality (*40*). Specifically, a recent transcriptomic study in the pharaoh ant, *Monomorium pharaonis*, reported a large suite of differentially expressed genes in larvae and caregiving nurses across larval development (*41*). The authors refer to this phenomenon as the “social interactome” of this species, a molecular signature of social interactions between larvae and nurses. In the context of our study, note that dopamine-related genes and *PebIII* were also among the differentially expressed genes in larvae and nurses in *M. pharaonis* [see the S1 dataset of (*41*)].

We went a step beyond the correlational description of the social interactome (*41*) in earwigs and also controlled for confounding developmental effects for the genes in offspring. We experimentally manipulated the presence and absence of posthatching parent-offspring interactions and characterized the social functions of the two genes showing the most convincing signature of differential coexpression using RNAi knockdown experiments. On the basis of this strictly experimental and result-driven approach, we could test the DGE and IGE of the two candidate genes *Th* and *PebIII* on behavior and fitness and assign selfish versus altruistic function to their expression. Only together with these functional results were we able to interpret the differential coexpression in mothers and nymphs as at least partly shaped by coadaptation.

### Evolutionarily conserved dopaminergic pathway from insects to human

Our study adds to the view that the dopaminergic pathway may be evolutionarily conserved in the context of parent-offspring interaction from insects to mammals, including primates and humans. Regarding the *Th* gene for which we showed evidence of reciprocally altruistic effects in earwigs, it was expressed more in the FC treatment in both mothers and offspring. In addition to *Th* per se, we also found higher expression of other genes related to dopamine synthesis and degradation pathways in caring mothers, suggesting enhanced dopamine activity in the central nervous system of earwigs (*18*, *19*) but suppressed dopamine activity in the reproductive system (*20*–*22*). The dopaminergic pathway was formerly associated with parenting: In rat mothers, dopamine levels were associated with pup licking and grooming behavior (*42*); in vervet monkeys and humans, variable number tandem repeats in exon III of the dopamine receptor *DRD4* gene were associated with parent-offspring interactions (*43*–*46*). It is likely that the behavioral difference in vervet monkey and human mothers and offspring was due to differential expression of various alleles or different receptor sensitivity to dopamine resulting from the allelic polymorphism of *DRD4*. This pathway also appears to be crucial to the simpler and nonobligate mother-offspring interaction in earwigs, which suggests an ancestral or convergent function of dopamine in the evolution of the social bond between parents and offspring.

### Functionally co-opted *PebIII* in social evolution

Unlike the conserved dopamine, the *PebIII* gene, for which we showed evidence of reciprocally selfish effects, may be functionally co-opted along the trajectory of social evolution from solitary to subsocial and to eusociality. Its expression effects vary from direct control of offspring development to social regulation of development and additional control of maternal reproduction and to reproductive caste differentiation: In the solitary *Drosophila*, larval development was associated with *PebIII* expression in larvae through a DGE (*47*); in earwigs, we found that nymph development was influenced by *PebIII* expression in mothers through an IGE, and maternal expression of *PebIII* governs female reproduction in earwigs via a DGE. In the eusocial termite *Reticulitermes flavipes*, two transcripts of this gene are expressed caste specifically between sterile soldiers and reproductive alate (*48*). Although *PebIII* consistently influences offspring development in solitary *Drosophila* and subsocial earwigs, the regulation seemingly shifted from direct control by the offspring to indirect control by the parent. The function on female reproduction is similar in subsocial earwigs and eusocial termites but diverged from single-gene determination to two transcripts of the same gene with potential neofunctionalization or subfunctionalization between castes. Thus, our results on *PebIII* might have captured an intermediate functional state of this gene between solitary and eusocial species.

### Influence on parental reproductive investment

A long-standing different question in the literature on parent-offspring conflict is whether genes expressed in parents or offspring control reproductive investment (*49*). Our results in earwigs provide direct evidence for a compromise with partial control by genes expressed in both parent and offspring. Whether or not a female produced a second clutch was under offspring control and was modulated by *Th* expression in nymphs. However, how much the females invested in their future clutch relative to their current clutch was under maternal control and was influenced by *PebIII* expression in females.

**Fig. 6:**
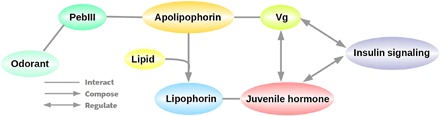
A hypothesized molecular pathway for a link between *PebIII* expression and reproduction. PebIII, an OBP, may play a role in chemical communication between mothers and offspring. PebIII indirectly interact with Vg and JH via apolipophorin and lipophorin (*25*). Insulin signaling, JH, and Vg interplay with each other and regulate maternal reproduction (*26*, *27*, *29*).

To elucidate how *PebIII* expression may influence maternal reproduction through a DGE in the subsocial earwigs, we found differential expression of genes related to insulin signaling, JH, and Vg, which consistently suggested suppressed insulin signaling and JH but elevated Vg in the FC treatment. These results are in line with previous results on caregiving in other insect systems such as burying beetles, ants, and bees (*26*, *27*, *30*, *34*, *35*) and provide indirect evidence for an evolutionary link between parenting genes and genes shaping eusociality (*50*). A graphical summary for this potential link between *PebIII*, insulin signaling, JH, and Vg is shown in Fig. 6.

In conclusion, it is a general expectation that the social bond between parents and their offspring is shaped by both altruistic and selfish behavior reflecting a compromise of their respective evolutionary interests (*1*). However, evolutionary theory ultimately relies on genetic or genomic support to demonstrate these tendencies. We used predictions of coadaptation theory and identified two genes that were differentially coexpressed in mothers and nymphs between treatments with or without posthatching parental care and mother-nymph interactions, *Th* and *PebIII*, with such distinct social functions. Neither *Th* nor *PebIII* was altruistic or selfish in a classical sense because both genes had a comparable function when expressed in mothers and offspring. These reciprocally altruistic and reciprocally selfish gene functions are peculiar because the gene’s fitness loss or gain during one life stage may at least partly be offset by its gain or loss during the other life stage. We envision that differential coexpression and reciprocally altruistic or selfish function are signatures of genes underlying parent-offspring coadaptation and may reflect a mechanism of co-regulation potentially through allelic variation in their cis-regulatory region. We predict that genes with differential coexpression and reciprocally altruistic or selfish function may be typical among genes underlying the social bond between parents and offspring and possibly also among caregivers and care recipients in other social systems.

## MATERIALS AND METHODS

### RNA-seq experiment

#### Experimental design and behavioral manipulation

The earwigs were maintained in the laboratory as previously described (*2*). A total of 90 randomly picked mated females from the breeding stocks were randomly assigned to three experimental treatments with 30 females per treatment. Females assigned to the NC treatment were isolated from their first clutch 1 day after oviposition, which is typically the time when the clutch is complete. These females were then kept in a new petri dish for 6 days without food, as is natural during the period of EC, and then euthanized for RNA extraction. Females assigned to the EC treatment tended their eggs for 20 days to ensure maximal duration of EC while avoiding any interaction with hatched nymphs. Hatching typically occurs after 21 to 30 days (*2*). The eggs were allowed to hatch, and the nymphs were kept with food for 6 days. On day 6 after hatching, three nymphs per clutch were sampled for RNA extraction. Females in the FC treatment tended their eggs until the eggs hatched and then fully interacted with the nymphs for 6 days. On day 6 after hatching, the females and three nymphs per clutch were sampled. Six days was chosen because mother-offspring interactions reach a peak at this time (*51*).

NC females were in a state where no maternal care could be expressed except for a maximum 1-day contact with eggs during oviposition. EC females could express care exclusively toward eggs, and only FC females could behaviorally interact with hatched offspring. With regard to offspring, the EC nymphs experienced no interactions with their mother, while the FC nymphs had such interactions for 6 days. Thus, differential gene expression between the FC and EC treatments in females and nymphs was assumed to be largely due to parent-offspring interactions. Differential expression between the EC and NC treatment in females was assumed to be largely due to egg attendance.

#### Sequencing

The insects were euthanized by exposure to high concentrations of petroleum ether before dissection. From females, the antennae, head, abdomen (without gut to avoid microbial contamination), and ovaries were sampled separately for RNA extraction to investigate tissue-specific variation in gene expression. Each maternal sample was a pool of the same tissue from five individuals to obtain sufficient RNA quantities. For nymphs, RNA was extracted from the whole body without dissection. Each nymph sample was a pool of five clutches with three nymphs per clutch to obtain sufficient RNA quantities. All samples were stored in RNAlater (QIAGEN) at −80°C.

A total of 84 samples were processed for RNA-seq, including six replicates of four female tissues across three treatments and nymph samples from two treatments: 6 × (4 × 3 + 2) = 84. NC nymphs were not used for sequencing because the hatching success of eggs without maternal care was too low (fig. S1 and table S1).

The RNA of each sample was extracted using the TRIzol protocol (Invitrogen), resulting in six replicates per tissue per treatment. The cDNA library was prepared and sequenced with paired-end 100-nucleotide reads on Illumina HiSeq. Each sample was indexed using an Illumina TruSeq kit. Samples from different treatments and tissues were evenly distributed among four multiplex lanes.

#### Bioinformatic and statistical analysis

An average of 18 million RNA-seq reads per sample was generated. The reads of each sample were mapped to a previously published earwig transcriptome (*14*) using the BWA-MEM algorithm in BWA version 0.7.8-r455 (*52*). SAMtools version 0.1.18 (*53*) was used to process sam files to bam format and to count mapped reads for each contig. Mapped reads with a mapping quality higher than MQ40 were processed for further analysis.

The initial statistical analysis of gene expression differences between experimental treatments was carried out using the edgeR package (*54*) in R. The RNA-seq data were TMM-normalized. Pairwise comparisons of each gene between the FC and EC treatment were performed for each female tissue and the nymph samples using exact negative binomial tests. To take into account multiple testing, we used an FDR correction as implemented in edgeR. Corresponding *P* values are denoted as *P*_FDR_.

Gene Ontology and Kyoto Encyclopedia of Genes and Genomes analysis were performed for differentially expressed genes with Blast2GO version 2.7.2 (*55*). A cutoff value was set at ×10^−6^ for the BLASTX search against the National Center for Biotechnology Information (NCBI) nonredundant nucleotide database and the SWISS-PROT database using the NCBI BLAST service.

### RNAi experiment

#### Experimental design

To test the effects of expression of the two candidate genes on females and nymphs, we carried out an RNAi experiment. To date, this is the first documented RNAi experiment performed on this nonmodel insect species. *Th* or *PebIII* was knocked down in separate treatments: knockdown in mothers (M−/O+), nymphs (M+/O−), and both (M−/O−). Three corresponding *YFP* treatments were used to control for the injection of exogenous double-stranded RNA. A total of 70 randomly mated female earwigs and their 1363 nymphs were randomly assigned to the experimental groups for behavioral and fitness assay. Clutch size was standardized to 20 nymphs in each family 1 day after hatching, except clutches that had less than 20 hatched nymphs. The sample size of each measured behavior and fitness trait was shown in table S5.

#### Synthesis of double-stranded RNA

The amplified sequences of each gene with a T7 promoter overhang at 5′ and 3′, respectively, were cloned from earwig cDNA to plasmids for storage and large-scale yields (table S3). Cloning was confirmed by sequencing the PCR product of each target gene from the plasmids. Double-stranded RNA was synthesized using RiboMAX large-scale RNA synthesis system T7 (Promega). The final concentration of double-stranded RNA used for injection was 6 μg/μl for each gene. The mothers were injected with 2 μl, and 20 nymphs from each family were injected with a total of 2 μl of double-stranded RNA on day 4 after the nymphs hatched. For RNAi injection, we used a CellTram air microinjector (Eppendorf) and borosilicate capillaries (Harvard) processed with a P-1000 Micropipette Puller (Sutter Instruments). Before injection, the earwigs were exposed to low concentrations of petroleum ether (Sigma-Aldrich, #77379) vapor for sedation.

#### Validation of knockdown

The double-stranded RNA was injected in the abdomen of mothers and offspring on day 4 after the nymphs hatched. The knockdown effects were initially validated with RT-qPCR after injection using three technical replicates per gene per sample. Each maternal sample was the head of a female. Each nymph sample was a pool of the whole body of three nymphs from the same clutch. Maternal samples included three *Th-*injected mothers, four *PebIII-*injected mothers, and two *YFP-*injected mothers. Offspring samples include three *Th-*injected nymphs, three *PebIII-*injected nymphs, and four *YFP-*injected nymphs. Both maternal and nymph samples were collected 3 days after injection. RNA was extracted using the TRIzol-LS reagent (Ambion). The cDNA libraries were synthesized using the GoScript Reverse Transcriptase system (Promega). The qPCR was run in triplicates on the Applied Biosystems 7500 Fast platform using an EvaGreen 2X qPCR Master Mix reagent (Biotium). Expression levels were calculated using ΔΔCt. The calibration was done separately for mothers and offspring. Results are shown in fig. S2.

#### Behavior and fitness assays

The developmental and reproductive variables were quantified following the standard protocol used in a previous study in this species (*2*). The rate of offspring development was quantified as the number of days from hatching until the first nymph of a family reached the second juvenile instar and nymph survival as the proportion of surviving nymphs 3 days after injection divided by the number of nymphs hatched.

Food provisioning was recorded during observation sessions under red light (earwigs are nocturnal) of 1 hour each across three consecutive days after injection, which was on days 5, 6, and 7 after the nymphs hatched. Observations were started 15 min after setup to calm down the animals. The occurrence of mouth-to-mouth contact between the female and one of her nymphs was treated as a binomial trait (yes/no). The observer was blind to experimental treatments. For quantitative traits, the average values across the three consecutive observation sessions were used in the analysis. For binary traits, at least one event across the three sessions counted as “yes.” The future reproduction of females was assessed by (i) noting whether a second clutch was produced within 60 days of hatching of the first clutch and by (ii) counting the relative size of second clutches as the proportion of eggs in the second clutch over the sum of eggs in the first and second clutch.

The survival of 70 mothers and their 1363 offspring was quantified 3 days after injection (table S6). It was difficult to tell whether they died immediately after injection because both mothers and nymphs were sedated before injection. Seven females died during the 3 days and had to be excluded from the analysis because no behavior or future reproduction was measurable. Nymphs from all 70 families were used for the quantification of survival because the mortality due to injection of double-stranded RNA was captured in our *YFP* sham treatments to which target gene treatments were compared.

#### Statistical analysis for behavior and fitness assay

The effects of the RNAi treatments on measured traits were tested using GLMs (tables S1 and S2). In the GLMs, main effects consist of fixed factors [maternal treatment effect, offspring treatment effect, and gene effect (target gene versus *YFP*)] and covariate (“oviposition date”). Interactive effects comprise the maternal treatment–by–gene interaction and offspring treatment–by–gene interaction. We used a backward model simplification procedure, eliminating effects sequentially according to their *P* value when *P* > 0.1, starting with the interaction terms and largest *P* values. When no interactive effect could be further removed, we continued with nonsignificant main effects, starting from the largest *P* value, provided that it was not part of an interactive effect still in the model. Continuous dependent variables were modeled using a Gaussian error distribution. Discrete or proportional dependent variables [food provisioning (yes/no), second clutch (yes/no), and relative size of second clutch and survival] were analyzed using a binomial error distribution and a logit link. Effects of the candidate gene are detectable as deviation from the *YFP* side effects as significant interactions between the factor gene and one of the treatment factors. Statistical analyses were carried out using R version 3.1.1; tests were two-tailed with a significance threshold of α = 0.05.

## Supplementary Material

http://advances.sciencemag.org/cgi/content/full/6/1/eaaw0070/DC1

Download PDF

Movie S1

Data file S1

Data file S2

Data file S3

Data file S4

Data file S5

The genetic mechanism of selfishnes and altruism in parent-offspring coadaptation

## SUPPLEMENTARY MATERIALS

Supplementary material for this article is available at http://advances.sciencemag.org/cgi/content/full/6/1/eaaw0070/DC1 Fig. S1. Hatching success of 5073 total eggs in RNA-seq experiment. Fig. S2. RT-qPCR validation for *Th* and *PebIII* knockdown. Fig. S3. Nonsignificant behavior and fitness results for *Th* and *PebIII* knockdown. Table S1. Hatching success of 5073 total eggs in RNA-seq experiment. Table S2. GLM results on behavior and fitness for *Th* and *PebIII* knockdown. Table S3. Nonsignificant GLM results of behavior and fitness assay for *Th* and *PebIII* knockdown. Table S4. Primers for double-stranded RNA synthesis. Table S5. Sample sizes in the behavioral and fitness assay of the RNAi experiment. Table S6. Mortality of mothers and offspring in RNAi experiment. Movie S1. Food provisioning in earwigs. Data file S1. List of genes responsive to parent-offspring interaction in earwig mothers’ antennae. Data file S2. List of genes responsive to parent-offspring interaction in earwig mothers’ head. Data file S3. List of genes responsive to parent-offspring interaction in earwig mothers’ abdomen. Data file S4. List of genes responsive to parent-offspring interaction in earwig mothers’ ovaries. Data file S5. List of genes responsive to parent-offspring interaction in earwig offspring.

## Appendix

View/request a protocol for this paper from *Bio-protocol*.
