# Supplementary material for: The genetic mechanism of selfishness and altruism in parent-offspring coadaptation
Source: Sci Adv. 2020 Jan 3;6(1):eaaw0070. doi: 10.1126/sciadv.aaw0070 (PMC6941917; doi:10.1126/sciadv.aaw0070)
Supplement: http://advances.sciencemag.org/cgi/content/full/6/1/eaaw0070/DC1 [file supp_6_1_eaaw0070__index.html]

Science Advances | Science AdvancesAAASSearchScience AdvancesMenu

## Supplementary Materials

**The PDFset includes:**

- Fig. S1. Hatching success of 5073 total eggs in RNA-seq experiment.
- Fig. S2. RT-qPCR validation for *Th* and *PebIII* knockdown.
- Fig. S3. Nonsignificant behavior and fitness results for *Th* and *PebIII* knockdown.
- Table S1. Hatching success of 5073 total eggs in RNA-seq experiment.
- Table S2. GLM results on behavior and fitness for *Th* and *PebIII* knockdown.
- Table S3. Nonsignificant GLM results of behavior and fitness assay for *Th* and *PebIII* knockdown.
- Table S4. Primers for double-stranded RNA synthesis.
- Table S5. Sample sizes in the behavioral and fitness assay of the RNAi experiment.
- Table S6. Mortality of mothers and offspring in RNAi experiment.
- Legend for movie S1
- Legends for data files S1 to S5

Download PDF

**Other Supplementary Material for this manuscript includes the following:**

- Movie S1 (.mp4 format). Food provisioning in earwigs.
- Data file S1 (Microsoft Excel format). List of genes responsive to parent-offspring interaction in earwig mothers’ antennae.
- Data file S2 (Microsoft Excel format). List of genes responsive to parent-offspring interaction in earwig mothers’ head.
- Data file S3 (Microsoft Excel format). List of genes responsive to parent-offspring interaction in earwig mothers’ abdomen.
- Data file S4 (Microsoft Excel format). List of genes responsive to parent-offspring interaction in earwig mothers’ ovaries.
- Data file S5 (Microsoft Excel format). List of genes responsive to parent-offspring interaction in earwig offspring.

**Files in this Data Supplement:**

- Adobe PDF - aaw0070\_SM.pdf
