# Supplementary material for: The genetic mechanism of selfishness and altruism in parent-offspring coadaptation
Source: Sci Adv. 2020 Jan 3;6(1):eaaw0070. doi: 10.1126/sciadv.aaw0070 (PMC6941917; doi:10.1126/sciadv.aaw0070)
Supplement: Download PDF [file aaw0070_SM.pdf]

Science Advances
AAAS

advances.sciencemag.org/cgi/content/full/6/1/eaaw0070/DC1

Supplementary Materials for

The genetic mechanism of selfishness and altruism in parent-offspring coadaptation

Min Wu*, Jean-Claude Walser, Lei Sun, Mathias Kölliker*

*Corresponding author. Email: miniversewu@gmail.com (M.W.); mathias.koelliker@bs.ch (M.K.)

Published 3 January 2020, *Sci. Adv.* **6**, eaaw0070 (2020)
DOI: 10.1126/sciadv.aaw0070

The PDF file includes:

Fig. S1. Hatching success of 5073 total eggs in RNA-seq experiment.
Fig. S2. RT-qPCR validation for *Th* and *PebIII* knockdown.
Fig. S3. Nonsignificant behavior and fitness results for *Th* and *PebIII* knockdown.
Table S1. Hatching success of 5073 total eggs in RNA-seq experiment.
Table S2. GLM results on behavior and fitness for *Th* and *PebIII* knockdown.
Table S3. Nonsignificant GLM results of behavior and fitness assay for *Th* and *PebIII* knockdown.
Table S4. Primers for double-stranded RNA synthesis.
Table S5. Sample sizes in the behavioral and fitness assay of the RNAi experiment.
Table S6. Mortality of mothers and offspring in RNAi experiment.
Legend for movie S1
Legends for data files S1 to S5

Other Supplementary Material for this manuscript includes the following:

(available at advances.sciencemag.org/cgi/content/full/6/1/eaaw0070/DC1)

Movie S1 (.mp4 format). Food provisioning in earwigs.
Data file S1 (Microsoft Excel format). List of genes responsive to parent-offspring interaction in earwig mothers' antennae.
Data file S2 (Microsoft Excel format). List of genes responsive to parent-offspring interaction in earwig mothers' head.
Data file S3 (Microsoft Excel format). List of genes responsive to parent-offspring interaction in earwig mothers' abdomen.
Data file S4 (Microsoft Excel format). List of genes responsive to parent-offspring interaction in earwig mothers' ovaries.
Data file S5 (Microsoft Excel format). List of genes responsive to parent-offspring interaction in earwig offspring.

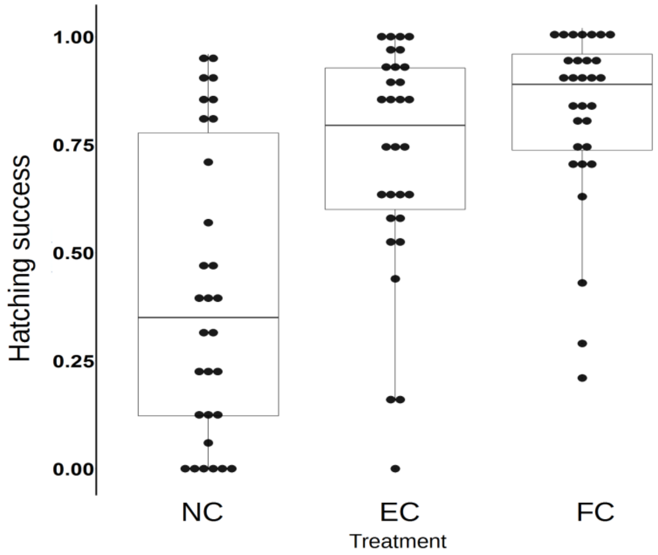

Fig. S1. Hatching success of 5073 total eggs in RNA-seq experiment. NC is no-care treatment, EC is egg-care treatment, and FC is full-care treatment.

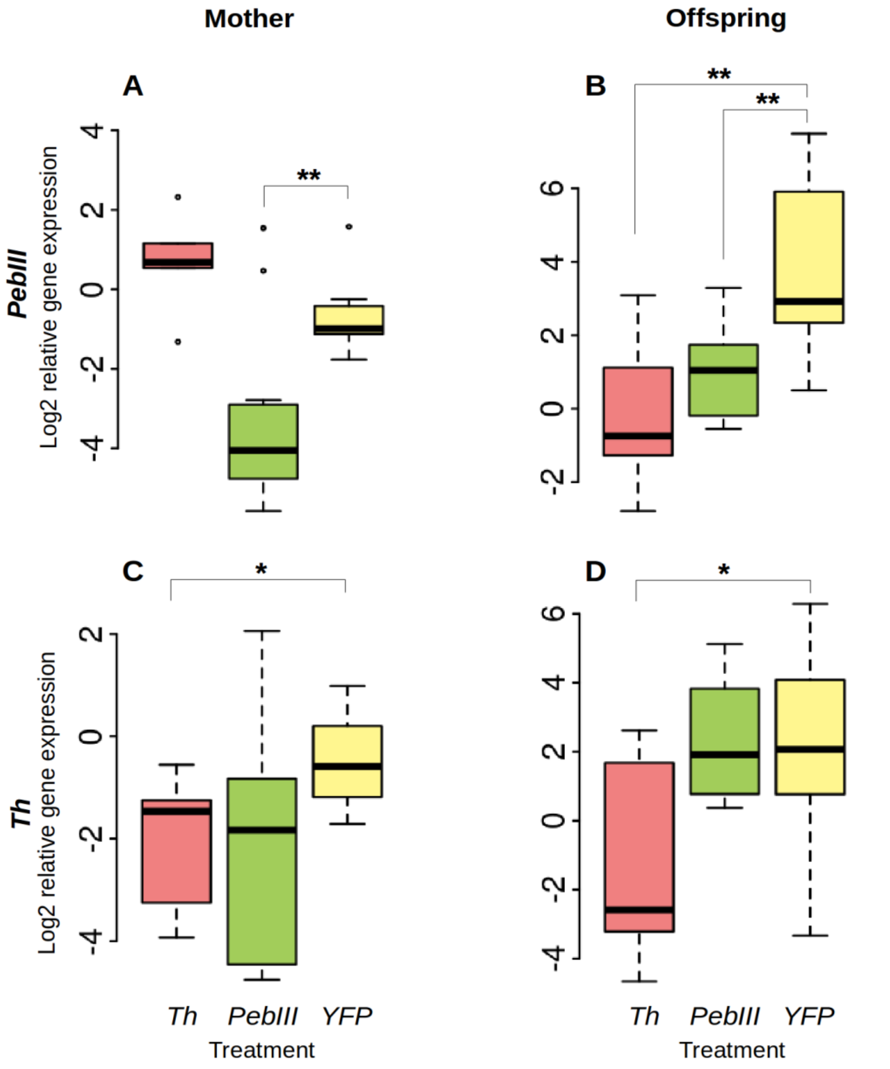

Fig. S2. RT-qPCR validation for *Th* and *PebIII* knockdown. Comparing to the corresponding controls with *YFP* ds-RNA injection, *PebIII* expression was reduced in mothers and in the offspring with *PebIII*-injection as expected (Wilcoxon test, $P_{mothers}$=0.0097, $P_{offspring}$=0.0091). *Th* expression in mothers and in the offspring with *Th* ds-RNA injection was also lower than the corresponding *YFP*-injected controls (Wilcoxon test, $P_{mothers}$=0.035, $P_{offspring}$=0.038). *PebIII* expression was also reduced in the offspring with *Th*-injection (wilcoxon test, P=0.0059). P<0.05*, P<0.01**.

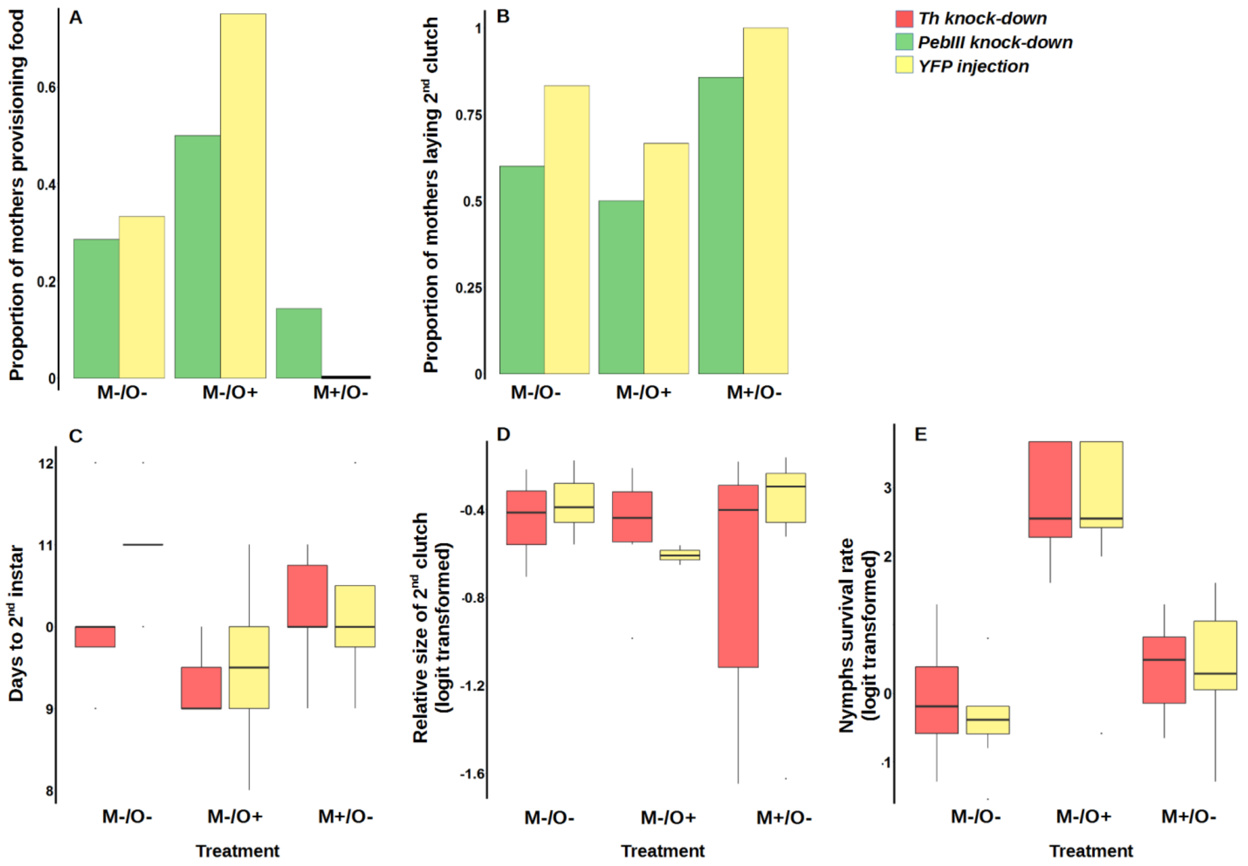

Fig. S3. Nonsignificant behavior and fitness results for *Th* and *PebIII* knockdown. (A) and (B) are results for *PebIII* knock-down. (C) to (E) are results for *Th* knock-down. In (D), the relative size of the 2nd clutch was calculated based on first and second clutch egg-numbers; In (A) and (B), frequencies are shown, and (C) to (E) are box-plots with median, interquartile range (box), and 1.5x interquartile range (whiskers). Target genes were knocked-down in three treatments: only in mothers (M-/O+), only in offspring (M+/O-), and in both (M-/O-). Three corresponding treatments of *YFP* were used to control for the injection of exogenous double-stranded RNA.

Table S1. Hatching success of 5073 total eggs in RNA-seq experiment. FC**:** full-care treatment, EC: egg-care treatment, NC: no-care treatment. Five out of six NC-nymph samples contained at least one clutch with zero hatching success. And one NC-nymph sample contained two clutches with zero hatching success. Therefore, nymphs sample from the NC treatment were not processed for RNA-Seq sequencing.

Treatment	Total eggs	Hatched nymphs	Hatch Success
FC	1678	1399	0.83
EC	1705	1263	0.74
NC	1690	726	0.43

Table S2. GLM results on behavior and fitness for *Th* and *PebIII* knockdown. We used generalized linear models (GLM) to compare effects of target-gene knock-down to side-effects due to *YFP* injections in mothers, offspring or both on our measures of behavior, reproduction, development and survival. In the models, we defined the "gene", "maternal-treatment" and "offspring-treatment" as fixed factors. Date of oviposition was added as covariate. The interaction between "gene" and "maternal-treatment" or between "gene" and "offspring-treatment" tested the direct genetic effect (DGE) or the indirect genetic effect (IGE) of a target gene on the dependent trait over and above the injection of double-stranded RNA. P<0.05*, P<0.01**, P<0.001***.

Behavior and fitness consequences of *Th*						
**Maternal food provision** *(binary trait: Yes/No, binomial distribution, logit link)*						
	Df	Deviance Resid.	Df	Resid. Dev	Pr(>Chi)	
NULL			40	51.22		
Gene	1	0.23	39	50.99	0.63	
Maternal-treatment	1	0.49	38	50.50	0.48	
Gene:Maternal-treatment	1	8.15	37	42.35	0.0043	**
Rejected term:						
Oviposition	1	0.02	39	51.20	0.88	
Offspringl-treatment	1	1.25	37	49.72	0.26	
Gene:Offspringl-treatment	1	0.70	34	40.58	0.40	
**Likelihood of maternal future reproduction** *(binary trait: Yes/No, binomial distribution, logit link)*						
	Df	Deviance Resid.	Df	Resid. Dev	Pr(>Chi)	
NULL			35	32.44		
Gene	1	0.57	34	31.87	0.45	
Offspring-treatment	1	0.44	33	31.43	0.51	
Gene:Offspring-treatment	1	4.36	32	27.07	0.037	*
Rejected term:						
Oviposition	1	2.21	34	30.23	0.14	
Maternal-treatment	1	2.07	31	27.58	0.15	
Gene:Maternal-treatment	1	2.50	30	25.08	0.11	

Behavior and fitness consequences of *PebIII*							
**Offspring development** *(gaussion distribution, identity link)*							
	Df	Deviance Resid.	Df	Resid. Dev	F	Pr(>F)	
NULL			42	90.74			
Oviposition	1	4.84	41	85.90	4.08	0.05	.
Gene	1	4.86	40	81.04	4.10	0.05	.
Offspring-treatment	1	21.61	39	59.43	18.23	0.00013	***
Maternal-treatment	1	1.23	38	58.20	1.04	0.31	
Gene:Maternal-treatment	1	14.33	37	43.87	12.09	0.0013	**
Rejected term:							
Gene:Offspringl-treatment	1	1.47	37	56.73	1.24	0.27	

**Relative investment in maternal future reproduction** *(overdispersed binomial distribution, logit link)*						
	Df	Deviance Resid.	Df	Resid. Dev	Pr(>Chi)	
NULL			25	37.02		
Gene	1	0.40	24	36.61	0.53	
Offspring-treatment	1	7.27	23	29.35	0.0070	**
Maternal-treatment	1	1.49	22	27.86	0.22	***
Gene:Maternal-treatment	1	5.69	21	22.16	0.017	*

	Df	Deviance Resid.	Df	Resid. Dev	Pr(>Chi)	
Rejected term:						
Oviposition	1	0.00	24	35.86	0.96	
Gene:Offspringl-treatment	1	1.07	20	24.37	0.30	

Offspring survival rate *(overedispersed binomial distribution, logit link)*

	Df	Deviance Resid.	Df	Resid. Dev	Pr(>Chi)	
NULL			45	125.37		
Oviposition	1	5.73	44	119.64	0.017	*
Gene	1	0.01	43	119.64	0.94	
Offspring-treatment	1	71.67	42	47.97	<0.0001	***
Maternal-treatment	1	3.58	41	44.39	0.059	.
Gene:Offspring-treatment	1	4.34	40	40.05	0.037	*
Rejected term:						
Gene:Maternal-treatment	1	1.06	39	39.35	0.30	

Table S3. Nonsignificant GLM results of behavior and fitness assay for *Th* and *PebIII* knockdown. We used generalized linear models (GLM) to compare effects of target-gene knock-down to effects of *YFP* injections in mothers, offspring or both on behavior, reproduction and development. In the models, we defined the “gene”, “maternal-treatment” and “offspring-treatment” as fixed factors. Date of oviposition was added as covariate. The interaction between “gene” and “maternal-treatment” or between “gene” and “offspring-treatment” tested the direct genetic effect (DGE) or the indirect genetic effect (IGE) of a target gene on the dependent trait over and above potential side-effects due to the injection of double-stranded RNA. P<0.05*, P<0.01**, P<0.001***.

Behavior and fitness consequences of *PebIII*

Maternal food provision *(binary trait: Yes/No, binomial distribution, logit link)*

	Df	Deviance Resid.	Df	Resid. Dev	Pr(>Chi)	
NULL			39	48.87		
Offspring-treatment	1	6.29	38	42.58	0.01	*
Rejected terms:						
Oviposition	1	2.18	38	46.69	0.14	
Maternal-treatment	1	2.53	36	36.87	0.11	
Gene	1	0.08	37	46.61	0.77	
Gene:Maternal-treatment	1	1.95	34	34.89	0.16	
Gene:Offspring-treatment	1	0.37	33	34.52	0.54	

Likelihood of maternal future reproduction *(binary trait: Yes/No, binomial distribution, logit link)*

	Df	Deviance Resid.	Df	Resid. Dev	Pr(>Chi)
NULL			34	37.63	
Rejected terms:					
Gene	1	2.40	33	35.23	0.12
Offspring-treatment	1	2.23	32	33.00	0.14
Maternal-treatment	1	2.22	31	30.78	0.14
Oviposition	1	0.03	33	37.60	0.87
Gene:Maternal-treatment	1	0.83	29	29.09	0.36
Gene:Offspring-treatment	1	0.23	28	28.86	0.63

Behavior and fitness consequences of *Th*

Offspring development *(gaussion distribution, identity link)*

	Df	Deviance Resid.	Df	Resid. Dev	F	Pr(>F)	
NULL			42	42.98			
Oviposition	1	2.59	41	40.39	3.14	0.084	.
Offspring-treatment	1	7.45	40	32.93	9.05	0.0045	**
Rejected terms:							
Gene	1	1.36	40	39.03	1.69	0.20	
Maternal-treatment	1	0.40	38	30.94	0.49	0.49	
Gene:Maternal-treatment	1	1.00	37	29.94	1.23	0.27	
Gene:Offspring-treatment	1	0.50	37	30.45	0.64	0.43	

Relative investment in maternal future reproduction *(overdispersed binomial distribution, logit link)*

	Df	Deviance Resid.	Df	Resid. Dev	Pr(>Chi)
NULL			28	31.12	
Rejected terms:					
Oviposition	1	2.20	27	28.92	0.14
Gene	1	0.59	26	27.85	0.44

Maternal-treatment	1	0.09	25	26.70	0.77	
Offspring-treatment	1	0.19	25	26.01	0.66	
Gene:Offspring-treatment	1	0.90	23	24.59	0.34	
Gene:Maternal-treatment	1	0.11	22	23.49	0.75	

Offspring survival rate *(overedispersed binomial distribution, logit link)*

	Df	Deviance Resid.	Df	Resid. Dev	Pr(>Chi)	
NULL			46	104.41		
Oviposition	1	6.67	45	97.74	0.010	**
Offspring-treatment	1	49.68	44	48.06	<0.0001	***
Maternal-treatment	1	5.43	43	42.63	0.020	*
Rejected terms:						
Gene	1	0.09	44	99.54	0.76	
Gene:Offspring-treatment	1	0.42	41	41.88	0.52	
Gene:Maternal-treatment	1	0.13	40	40.89	0.71	

Table S4. Primers for double-stranded RNA synthesis.

Gene	RNA Strand	Name	Primer sequence	Amplicon length
Th	1	TH_F	5'-CTG GGA CAC ATG CCA CTT CT-3	774
		TH_RT	5'-AAA GCG GCC GCT AAT ACG ACT CAC TAT AGG TCG TCA GTT TCC AGC TCC AC-3	
	2	TH_FT	5'-AAA GCG GCC GCT AAT ACG ACT CAC TAT AGG CTG GGA CAC ATG CCA CTT CT-3	774
		TH_R	5'-TCG TCA GTT TCC AGC TCC AC-3	
PebIII	1	PEB_F	5'-TTG GTT CTC TTC GCT GAG GC-3	333
		PEB_RT	5'-AAA GCG GCC GCT AAT ACG ACT CAC TAT AGG TCC AGT TGG GTC GTA TTT CTC TT-3	
	2	PEB_FT	5'-AAA GCG GCC GCT AAT ACG ACT CAC TAT AGG TTG GTT CTC TTC GCT GAG GC-3	333
		PEB_R	5'-TCC AGT TGG GTC GTA TTT CTC TT-3	
YFP	1	YFP_F	5'-TTC AGT GTT TCG CGC GTT ATC-3'	530
		YFP_RT	5'-AAA GCG GCC GCT AAT ACG ACT CAC TAT AGG TTC AGT GTT TCG CGC GTT ATC-3'	
	2	YFP_FT	5'-AAA GCG GCC GCT AAT ACG ACT CAC TAT AGG CAT ACC CAG GGT AAT ACC GGC-3'	530
		YFP_R	5'-CAT ACC CAG GGT AAT ACC GGC-3'	

Table S5. Sample sizes in the behavioral and fitness assay of the RNAi experiment. Among the initial 70 females used in RNAi experiment, seven died in three days after injection and were excluded from the analysis because no measures on reproduction and maternal behavior available. Eight females used in the behavior and fitness assays and one additional injected female were sampled for qPCR validation and thus, were counted as missing data for the traits of future reproduction. Only females laying a 2nd clutch were used for the trait of relative investment in 2nd clutch (*2*).

Gene	Treatment	Food provisioning	Likelihood of 2C	Development	Relative investment 2C/1C	Survival
PEB	M-/ O-	7	5	6	3	8
PEB	M-/ O+	8	6	8	3	8
PEB	M+/ O-	7	7	7	6	7
YFP	M-/ O-	6	6	6	5	7
YFP	M-/ O+	4	4	8	2	8
YFP	M+/ O-	8	8	8	7	8
TH	M-/ O-	8	5	8	3	9
TH	M-/ O+	7	6	7	6	7
TH	M+/ O-	8	8	6	6	8
**Total size**	**PEB+YFP**	40	36	43	26	46
	**TH+YFP**	41	37	43	29	47

Table S6. Mortality of mothers and offspring in RNAi experiment. Among the 70 females, 7 died in three days after injection. Among the 1363 nymphs in RNAi experiment, 451 out of the 902 injected, and 28 out of 461 untreated died in three days after injection.

Gene	Treatment	Mother				Offspring			
		Survival	Total	Live	Dead	Survival	Total	Live	Dead
PEB	M-/ O-	0.88	8	7	1	0.43	160	69	91
PEB	M-/ O+	1	8	8	0	0.98	160	157	3
PEB	M+/ O-	1	7	7	0	0.50	135	67	68
YFP	M-/ O-	0.86	7	6	1	0.41	139	57	82
YFP	M-/ O+	0.5	8	4	4	0.89	160	142	18
YFP	M+/ O-	1	8	8	0	0.58	159	93	66
TH	M-/ O-	0.89	9	8	1	0.48	180	86	94
TH	M-/ O+	1	7	7	0	0.95	141	134	7
TH	M+/ O-	1	8	8	0	0.61	129	79	50
**Total**		0.9	70	63	7	0.65	1363	884	479

Movie S1. Food provisioning in earwigs. An earwig mother surrounded by her offspring is feeding them mouth-to-mouth.

Data file S1. List of genes responsive to parent-offspring interaction in earwig mothers' antennae. Differentially expressed genes between full-care and egg-care treatments in the antennae of earwig mothers. Genes with negative log fold changes were up-regulated in full-care samples. Abbreviations: logFC for log fold changes of expression; logCPM for log read counts per million reads; FDR for false discovery rate adjusted P value. Gene for predicted function according to BLAST and Hit description showed details of the top BLAST hit for each gene.

Data file S2. List of genes responsive to parent-offspring interaction in earwig mothers' head. Differentially expressed genes between full-care and egg-care treatments in the head of earwig mothers. Genes with negative log fold changes were up-regulated in full-care samples. Abbreviations: logFC for log fold changes of expression; logCPM for log read counts per million reads; FDR for false discovery rate adjusted P value. Gene for predicted function according to BLAST and Hit description showed details of the top BLAST hit for each gene.

Data file S3. List of genes responsive to parent-offspring interaction in earwig mothers' abdomen. Differentially expressed genes between full-care and egg-care treatments in the abdomen of earwig mothers. Genes with negative log fold changes were up-regulated in full-care samples. Abbreviations: logFC for log fold changes of expression; logCPM for log read counts per million reads; FDR for false discovery rate adjusted P value. Gene for predicted function according to BLAST and Hit description showed details of the top BLAST hit for each gene.

Data file S4. List of genes responsive to parent-offspring interaction in earwig mothers' ovaries. Differentially expressed genes between full-care and egg-care treatments in the ovaries of earwig mothers. Genes with negative log fold changes were up-regulated in full-care samples. Abbreviations: logFC for log fold changes of expression; logCPM for log read counts per million reads; FDR for false discovery rate adjusted P value. Gene for predicted function according to BLAST and Hit description showed details of the top BLAST hit for each gene.

Data file S5. List of genes responsive to parent-offspring interaction in earwig offspring. Differentially expressed genes between full-care and egg-care treatments in earwig nymphs. Genes with negative log fold changes were up-regulated in full-care samples. Abbreviations: logFC for log fold changes of expression; logCPM for log read counts per million reads; FDR for false discovery rate adjusted P value. Gene for predicted function according to BLAST and Hit description showed details of the top BLAST hit for each gene.
